# Synthesis of Novel Nitro-Halogenated Aryl-Himachalene Sesquiterpenes from Atlas Cedar Oil Components: Characterization, DFT Studies, and Molecular Docking Analysis against Various Isolated Smooth Muscles

**DOI:** 10.3390/molecules29122894

**Published:** 2024-06-18

**Authors:** Youssef Edder, Issam Louchachha, Abdelmajid Faris, Mohamed Maatallah, Khalil Azzaoui, Mohammed Zerrouk, Mohamed Saadi, Lahcen El Ammari, Moha Berraho, Mohammed Merzouki, Brahim Boualy, Belkheir Hammouti, Rachid Sabbahi, Abdallah Karim, Mohammed M. Alanazi, Alicia Ayerdi Gotor, Larbi Rhazi

**Affiliations:** 1 Laboratory of Molecular Chemistry, Faculty of Sciences Semlalia, Cadi Ayyad University, BP 2390, Marrakech 40001, Morocco; louchachhaissam@gmail.com (I.L.); abdelmajid.faris@gmail.com (A.F.); m.maatallah@uca.ma (M.M.); karim@uca.ac.ma (A.K.); 2 Engineering Laboratory of Organometallic, Molecular Materials, and Environment, Faculty of Sciences, University Sidi Mohamed Ben Abdellah, Fes 30000, Morocco; k.azzaoui@yahoo.com (K.A.); zrkmed0650@gmail.com (M.Z.); 3 Euromed Research Center, Euromed Polytechnic School, Euromed University of Fes, Fes 30030, Morocco; hammoutib@gmail.com; 4 Laboratoire de Chimie Appliquée des Matériaux, Centre des Sciences des Matériaux, Faculty of Science, Mohammed V University in Rabat, Avenue Ibn Battouta, BP 1014, Rabat 10000, Morocco; m.saadi@um5r.ac.ma (M.S.); l.elammari@um5r.ac.ma (L.E.A.); 5 Laboratoire de Chimie des Substances Naturelles, Unité Associée au CNRST (URAC16), Faculty of Sciences Semlalia, Cadi Ayyad University, BP 2390, Marrakech 40001, Morocco; berrahomoha7@gmail.com; 6 Laboratory of Applied Chemistry Environment (LCAE-ECOMP), Faculty of Science Oujda, University Mohammed First, Oujda 60000, Morocco; moh.merzouki@gmail.com; 7 Multidisciplinary Research and Innovation Laboratory, Faculté Polydisciplinaire de Khouribga, Université Sultan Moulay Slimane de Beni-Mellal, Khouribga 23000, Morocco; b.boualy@gmail.com; 8 Research Team in Science and Technology, Higher School of Technology, Ibn Zohr University, Laayoune 70000, Morocco; r.sabbahi@uiz.ac.ma; 9 Department of Pharmaceutical Chemistry, College of Pharmacy, King Saud University, Riyadh 11451, Saudi Arabia; mmalanazi@ksu.edu.sa; 10 Institut Polytechnique UniLaSalle, AGHYLE, UP 2018.C101, UniLaSalle, 19 Rue Pierre Waguet, BP 30313, 60026 Beauvais, France; alicia.ayerdi-gotor@unilasalle.fr; 11 Institut Polytechnique UniLaSalle, Université d’Artois, ULR 7519, 19 Rue Pierre Waguet, BP 30313, 60026 Beauvais, France

**Keywords:** sesquiterpene, arylhimachalene, halogenation, nitration, DFT calculation, X-ray diffraction, molecular docking

## Abstract

We report the synthesis of two novel halogenated nitro-arylhimachalene derivatives: 2-bromo-3,5,5,9-tetramethyl-1-nitro-6,7,8,9-tetrahydro-5H-benzo[7]annulene (bromo-nitro-arylhimachalene) and 2-chloro-3,5,5,9-tetramethyl-1,4-dinitro-6,7,8,9-tetrahydro-5H-benzo[7]annulene (chloro-dinitro-arylhimachalene). These compounds were derived from arylhimachalene, an important sesquiterpene component of Atlas cedar essential oil, via a two-step halogenation and nitration process. Characterization was performed using ^1^H and ^13^C NMR spectrometry, complemented by X-ray structural analysis. Quantum chemical calculations employing density functional theory (DFT) with the Becke3-Lee-Yang-parr (B3LYP) functional and a 6-31++G(d,p) basis set were conducted. The optimized geometries of the synthesized compounds were consistent with X-ray structure data. Frontier molecular orbitals and molecular electrostatic potential (MEP) profiles were identified and discussed. DFT reactivity indices provided insights into the compounds’ behaviors. Moreover, Hirshfeld surface and 2D fingerprint analyses revealed significant intermolecular interactions within the crystal structures, predominantly H–H and H–O contacts. Molecular docking studies demonstrate strong binding affinities of the synthesized compounds to the active site of protein 7B2W, suggesting potential therapeutic applications against various isolated smooth muscles and neurotransmitters.

## 1. Introduction

**Figure 1:**
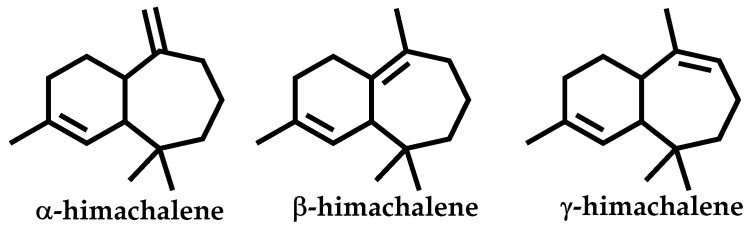
Himachalene isomers, the major components of Atlas cedar oil.

**Figure 2:**
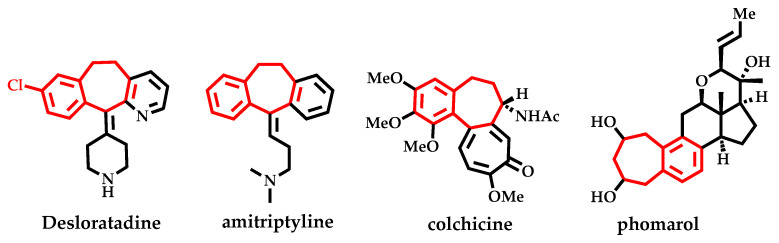
Pharmaceuticals and natural products with benzocycloheptane motif.

The essential oil of Atlas cedar, *Cedrus atlantica*, is essentially composed of sesquiterpenic hydrocarbons α-, β-, and γ-himachalene (Figure 1) and together they can make up to 70% of its contents [1]. This oil can be explored as a renewable feedstock for several industrial processes and pharmaceutical compounds. Arylhimachalene is another minor constituent of this oil that can easily be obtained by aromatizing the α-, β-, and γ-himachalene mixture [2]. Structurally, arylhimachalene is a natural benzocylcohetane; this structure motif is present in numerous important medicines (desloratadine [3] and amitriptyline [4]) (Figure 2) and natural products (colchicine and phomarol) (Figure 2). It is an important building block for the synthesis of biologically active compounds. Thus, we are interested in himachalenes, and particularly in arylhimachalene, as a readily available starting material for the synthesis of novel biologically active benzocycloheptanic derivatives of himachalenes. In fact, we have previously reported the synthesis and molecular docking analysis of trans-himachalol, a synthetic isomer of the naturally occurring sesquiterpene cis-himachalol; the synthesized compound has shown promising activities against various isolated smooth muscles and against different neurotransmitters [5]. Moreover, arylhimachalene derivatives have been previously reported to exhibit different biological activities [6,7,8].

Nitroarenes are a well-known compound with versatile applications from perfumes and pharmaceuticals to dyes and explosives [9,10,11]. They can be obtained by an electrophilic hydrogen substitution [12,13,14]. The reactivity of the aromatic compounds depends on the substituted groups. They can either activate or deactivate the arenes. Substituted groups can also act as directing agents, favoring one regioisomer over another [15].

In order to enhance the previously reported promising biological activities of himachalenes derivatives [6,7,8], herein, we report the syntheses and the structural characterization of two new halogenated nitro arylhimachalene: 2-bromo-3,5,5,9-tetramethyl-1-nitro-6,7,8,9-tetrahydro-5H-benzo[7]annulene (bromo-nitro-arylhimachalene) and 2-chloro-3,5,5,9-tetramethyl-1,4-dinitro-6,7,8,9-tetrahydro-5H-benzo[7]annulene (chloro-dinitro-arylhimachalene) using ^1^H and ^13^C NMR spectroscopy and X-ray crystallography. In addition, the electronic properties and chemical reactivity were evaluated based on the density functional theory (DFT). A Hirshfeld surface analysis was carried out in order to study intermolecular interactions in the synthesized crystals. Finally, molecular docking studies reveal that the synthesized compounds possess promising therapeutic potential for modulating various isolated smooth muscle tissues and neurotransmitter pathways.

## 2. Results and Discussion

2-bromo-1-ntitro-arylhimachalene (**4**) and 2-chloro-1,4-dintitro-arylhimachalene (**5**) were prepared from arylhimachalene in a two-step process. The first step is the halogenation of arylhimachalene, and the second step is the nitration of the halogenated products by a mixture of nitric acid and sulfuric acid.

### 2.1. Halogenation of Arylhimachalene

N-halosuccinimides are the first candidates for mild and safe aromatic halogenation [16,17]. However, N-halocussinimides are also used for benzylic halogenation [18], and their use may lead to competitive reactions. Otherwise, most of the alternative methods reported consist of the use of an oxidant and a hydrohalic acid or halide salt: K_2_S_2_O_8_/NH_4_Cl [19], TBHP (or H_2_O_2_)/HCl [20], HIO_3_/KBr [21], and H_2_O_2_/NH_4_I [22].

**Scheme 1:**
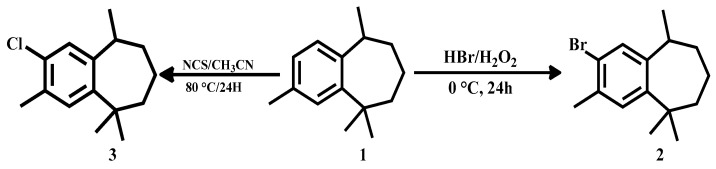
Halogenation of arylhimachalene.

The treatment of arylhimachalene with N-chlorosuccinimide (NCS) took place in acetonitrile at 80 °C [16] and led to the formation of chloro-arylhimachalene (**3**) with an 85% yield (Scheme 1). The absence of any benzylic chlorination products even with the use of two equivalents of NCS suggests that the reaction likely proceeds via an electrophilic substitution mechanism rather than a radical one [23].

The good results obtained for the chloration of arylhimachalene with NCS encouraged us to use N-bromosuccinimide (NBS) for the bromination. The use of NBS led to a total conversion but an unsatisfying selectivity due to the formation of other multibromated products like the (Z)-2,8-dibromo-9-(bromomethylene)-3,5,5-trimethyl-6,7,8,9-tetrahydro-5H-benzo[7]annulene that can be obtained using the same protocol [24]. To enhance selectivity, arylhimachalene was subjected to a HBr/H_2_O_2_ treatment. This approach was highly effective, achieving complete conversion of arylhimachalene with 100% selectivity for the target product 2 (Scheme 1).

### 2.2. Nitration of Arylhimachalane-Halogenated Derivatives

**Scheme 2:**
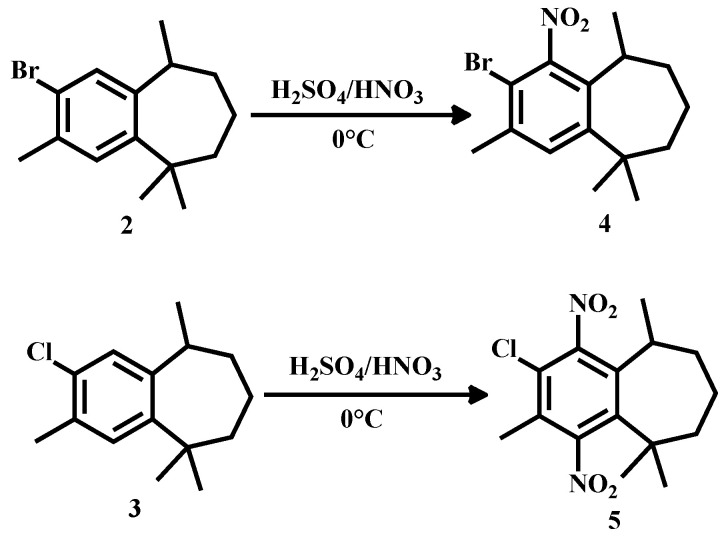
Nitration of bromo-arylhimachalene and chloro-arylhimachalene.

While treating the bromo-arylhimachalene with a mixture of nitric acid and sulfuric acid led to a total conversion, giving the nitro derivative (**4**) an 83% yield, treating chloro-arylhimachalene with the same acid mixture gave chloro-dinitro-arylhimachalene (**5**) instead of the mono-nitrated derivative with a 79% yield (Scheme 2).

### 2.3. Structural Description of the Compounds

#### 2.3.1. 2-Bromo-1-Ntitro-Arylhimachalene

**Figure 3:**
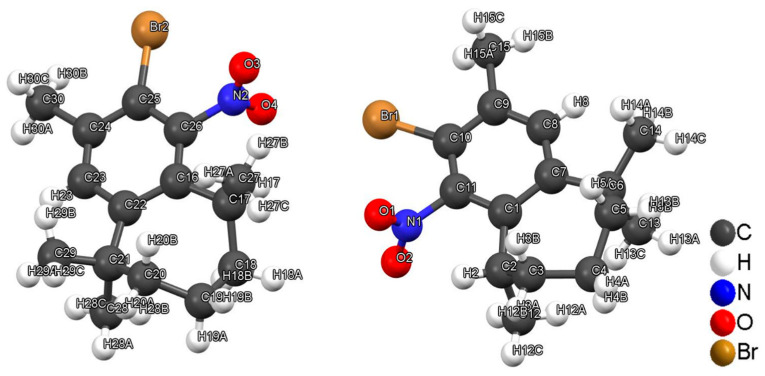
Molecular structure of 2-bromo-1-ntitro-arylhimachalene with the two molecules constituting the asymmetric unit.

**Figure 4:**
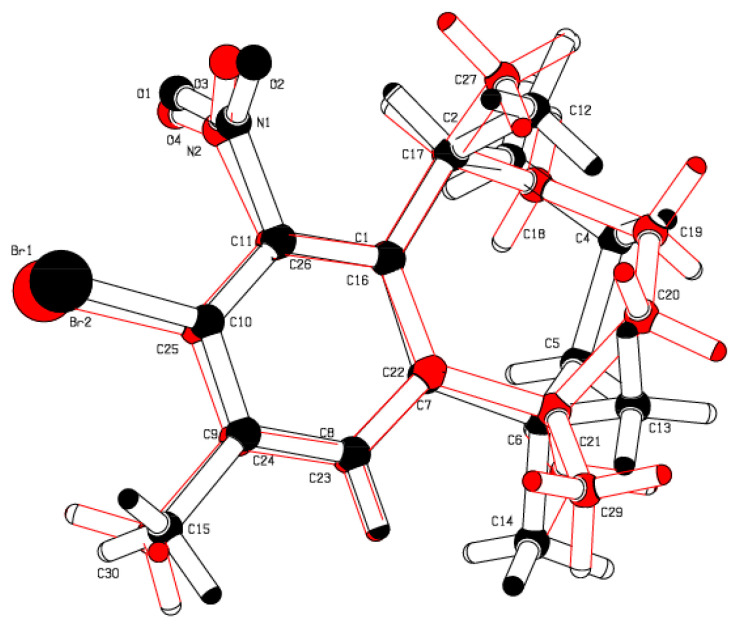
Automatic fit of the two crystallographically independent molecules.

**Figure 5:**
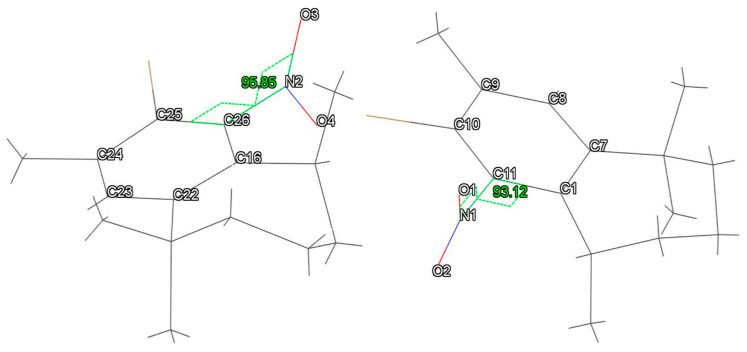
Dihedral angles between the planes formed by the nitro groups and benzene rings in the two independent molecules.

**Figure 6:**
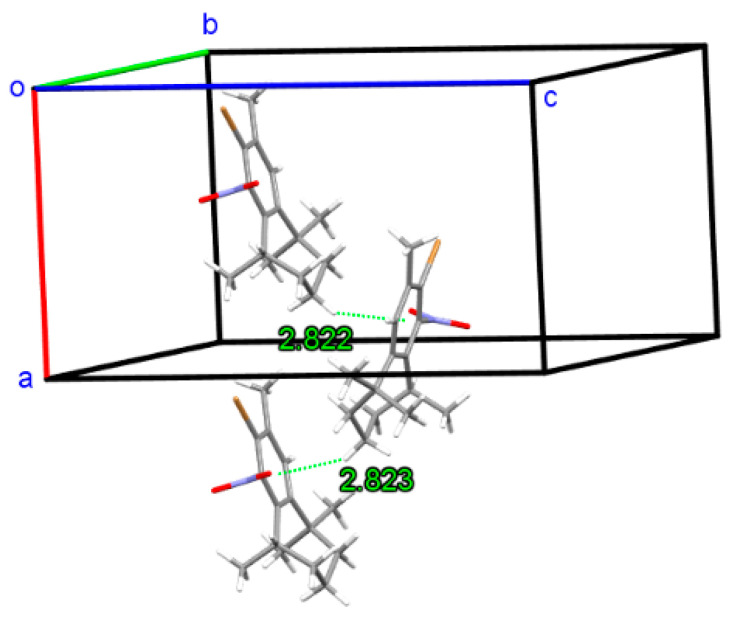
Interaction of C4—H4B…Cg1, where Cg1 denotes the centroid of the benzene ring.

**Figure 7:**
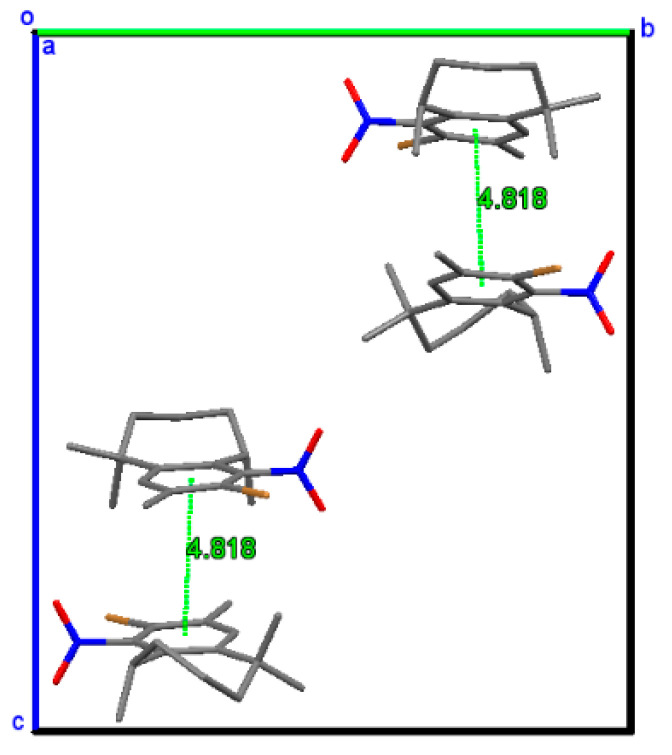
π–π stacking interactions between the benzene rings.

**Figure 8:**
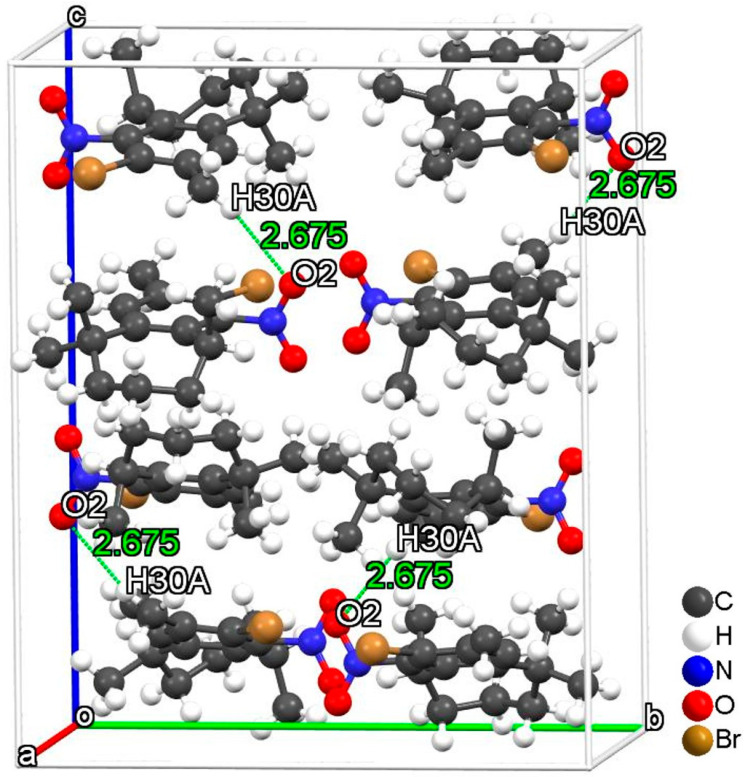
Molecule and its symmetry partner linked by C30–H30A···O2, representing a non-classical hydrogen bond. Symmetry codes: (i) −x + 1, y + 1/2, and −z + 3/2.

The compound crystallizes in the orthorhombic system with the P2_1_2_1_2_1_ space group. The molecular structure of this compound is illustrated in Figure 3. The asymmetric unit comprises two distinct molecules exhibiting different conformations, which represents the automatic fit of the two crystallographically independent molecules, as illustrated in Figure 4 [25]. Each molecule is built up from fused six- and seven-membered rings. In the first molecule (C1–C15), the seven-membered ring shows a chair conformation, whereas in the second molecule (C16–C30), the seven-membered ring displays a screw-boat conformation. The plane passing through the nitro group (O1O2N1C11) is almost perpendicular to the benzene ring (C1 to C11), as indicated by the value of the dihedral angle of (O1O2N1C11)/(C1 to C11), which is equal to 93.12°, while the plane passing through the nitro group (O3O4N2C26) is slightly offset from the perpendicular to the benzene ring, as shown by the value of the dihedral angle (O3O4N2C26)/(C16 to C26), which is equal to 95.85°; Figure 5 shows the two dihedral angles. Table 1 summarizes the crystallographic data, experimental details of data collection, and structure refinements. The crystal structure’s interlayer connectivity is maintained through hydrogen bonds (C30–H30A… O2^i^), involving H atoms from methyl groups on benzene rings and offset π…π interactions between aromatic rings. These interactions are illustrated in Figure 6 and Figure 7, with a centroid-to-centroid distance of 4.818 (4) Å. Additionally, the crystal structure exhibits C4–H4B… Cg1^ii^ interactions, where Cg represents the centroid of the benzene rings. Collectively, these interactions create a three-dimensional network, depicted in Figure 8.

#### 2.3.2. 2-Chloro-1:3-Dintitro-Arylhimachalene

**Figure 9:**
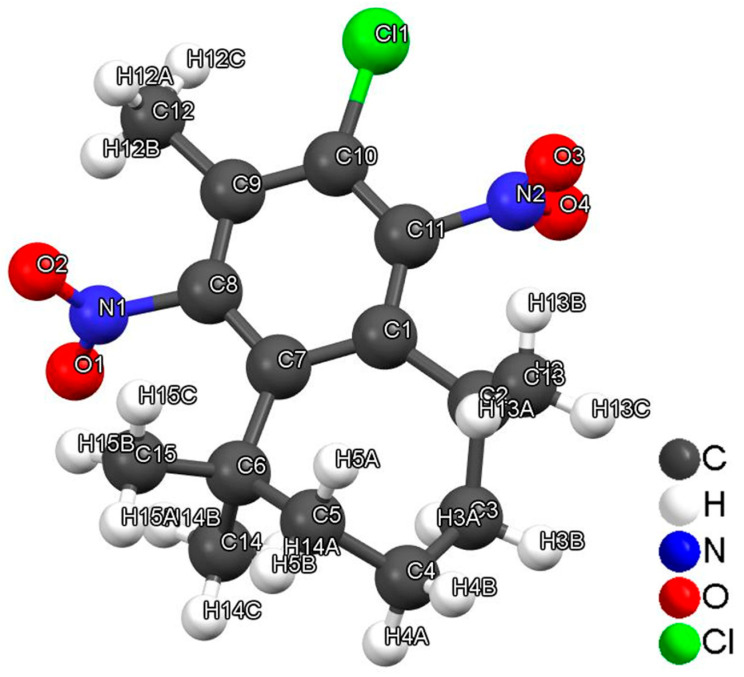
Molecular structure of 2-chloro-1,3-dintitro-arylhimachalene.

**Figure 10:**
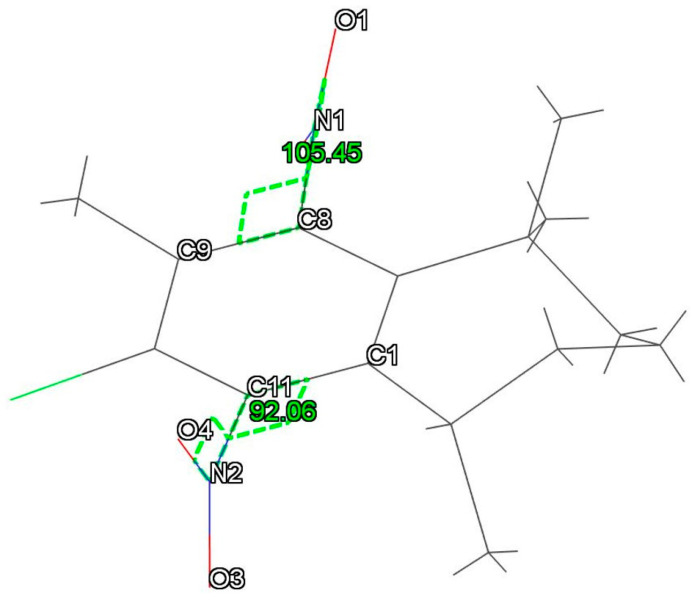
Dihedral angles between the planes formed by the nitro groups and benzene ring.

The synthesized compound C_15_H_19_ClN_2_O_4_ consists of two fused rings: a six-membered and seven-membered ring. The seven-membered ring adopts a screw-boat conformation characterized by the following parameters: QT = 0.9266 (2) Å, θ = 68.85 (2) °, φ2 = −40.54 (1) °, and φ3 = 166.87 (3) ° [26]. The presence of the Cl atom allows for the absolute configuration to be definitively determined as C2(S) [27]. The bond length of C10–Cl1 is 1.73 Å, which aligns well with the reported value of 1.738 (2) for a similar bond between a benzene ring and a chlorine atom [28]. The molecular structure is illustrated in Figure 9. The plane formed by the nitro group (O3O4N2C11) is nearly perpendicular to the benzene ring, with a dihedral angle of 92.06°, while the dihedral angle between the nitro group (O1O2N1C8) and the benzene ring is 105.45°. Figure 10 illustrates these two dihedral angles. The crystal packing does not exhibit any specific intermolecular interactions.

### 2.4. DFT Computation and Electronic Structure

**Figure 11:**
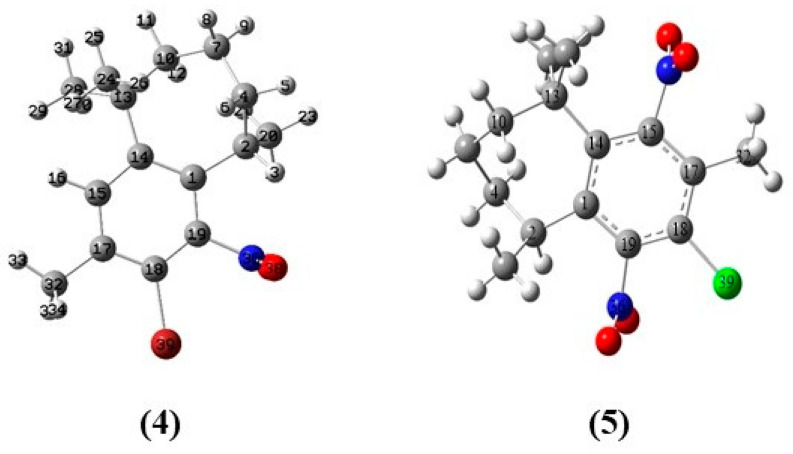
Optimized structures of compounds (**4**) and (**5**) obtained using DFT at the 6-31++G(d,p) basis.

To gain a deeper understanding of the electronic structure, the optimization of compounds **4** and **5** was performed using the DFT/B3LYP method, focusing on the singlet ground state. Figure 11 shows the optimized geometry of the two molecules, while Table 2 lists the selected structural parameters, bond lengths, bond angles, and torsion angles, with respect to the most stable conformer in the optimized structure and the single crystal.

The data presented in Table 2 show that the geometrical parameters for the synthesized compounds **4** and **5** exhibit a high degree of concordance between the experimental and theoretical results. The majority of the calculated values are in close alignment with the experimental data, demonstrating no notable discrepancies between the single crystal and optimized structures of these stable compounds (**4** and **5**). The computed geometrical parameters excellently coincide with the experimental data. However, in a few cases, slight mismatches can be attributed to phase differences between the experiment and computation, as well as to the absence of packing effects in the gas-optimized structure. Overall, these results indicate a strong concurrence between the experimental findings and those obtained through the DFT calculations.

### 2.5. Frontier Molecular Orbital (FMO) and Reactivity Parameter Analysis

The theoretical calculations yielded valuable insights into the energies of the highest occupied molecular orbital (HOMO) and the lowest unoccupied molecular orbital (LUMO). These frontier orbitals are crucial for demonstrating a molecule’s electron-donating or electron-accepting properties. Furthermore, they serve as a foundation for calculating various reactivity parameters according to Koopman’s theorem [29]. Global reactivity parameters are pivotal for determining the reactivity profiles of organic compounds, offering a deeper understanding of their chemical reactivity and kinetic stability, as well as their electronic behavior during chemical processes. Key parameters include the chemical potential (μ), chemical hardness (η), electronegativity (χ), and electrophilicity index (ω). The calculated values of these electronic quantities characterizing the molecular structures are listed in Table 3.

Compounds exhibiting a small energy difference between the HOMO and LUMO (ΔEgap) and high softness (σ) demonstrate heightened reactivity. This leads to stronger interactions when a target molecule with high HOMO energy acts as an electron donor, interacting with a molecule serving as an acceptor with lower LUMO energy.

The calculated energy gaps for the synthesized compounds **4** and **5** are 4.351 eV and 4.526 eV, respectively. These values suggest that the compounds exhibit low kinetic stability and high chemical reactivity, making them good precursors for further development and synthesis of more complex molecules.

Notably, compound **5** displays lower energy orbitals than compound **4**, indicating enhanced reactivity in the latter. This behavior is further supported by the observed values of chemical potential and hardness. Compound **4**, with a high electrophilicity index of 3.013 eV, is identified as a potent electrophile, thereby acting as a more effective electron acceptor than compound **5**.

**Figure 12:**
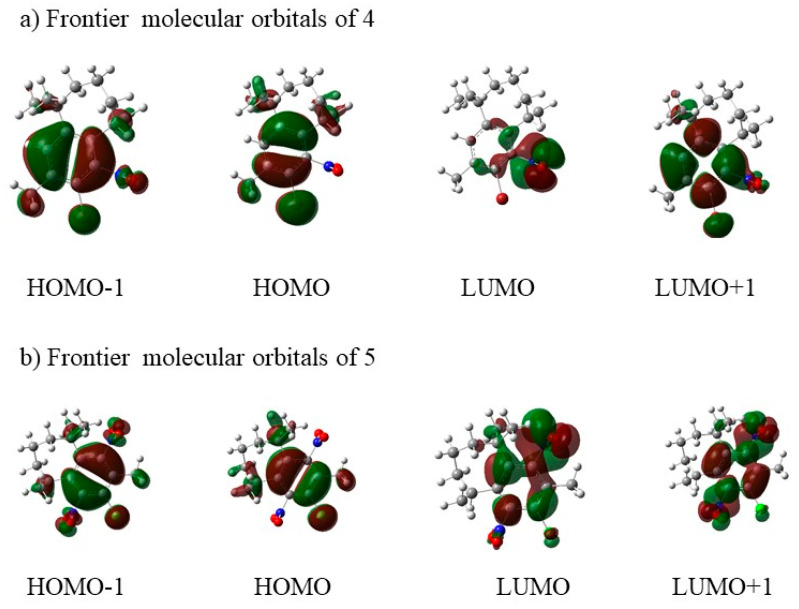
Density distributions of the frontier molecular orbitals for the studied compounds, calculated using the B3LYP/6-31G++ (d,p) level of theory.

For compound **4**, the HOMO is primarily localized on the two double bonds C1=C14 and C17=C18, as well as on the bromine atom. However, no electron density is observed on the C15 and C19 atoms. Conversely, the LUMO is predominantly located in the NO_2_ group. In the HOMO-1 orbital, the electron density is mainly concentrated on the phenyl ring, particularly on the double bonds C15=C17, C1=C19, and C18=C19, and to a lesser extent on the bromine atom (Figure 12). The molecular orbitals of the C1=C14 and C17=C18 double bonds remain devoid of any electron density. An orbital contribution investigation indicates that the composition of HOMO comes mainly from the orbitals of C1, C14, C17, and C18 and Br39 atoms. In contrast, the components of LUMO come mainly from the nitro moiety (Table 4).

In compound **5**, the HOMO orbitals are primarily situated on the benzene ring, specifically on the two double bonds C1=C14 and C16=C17, as well as on the chlorine atom (Figure 12). In contrast, the NO_2_ groups exhibit a lack of electron density. The composition of HOMO comes mainly from the atomic orbitals of C1, C14, C16, C17, and Cl 35. As for the LUMO orbital, it is located on the C14=C15 and C17=C18 bonds and on one of the two NO_2_ groups (Table 4).

### 2.6. Molecular Electrostatic Potential (MEP)

**Figure 13:**
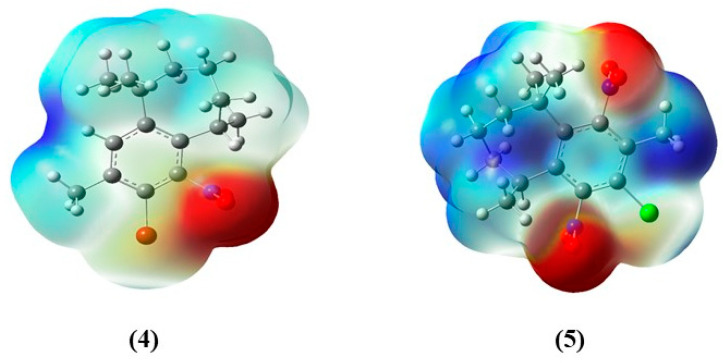
Molecular electrostatic potential surfaces of compounds **4** and **5**, calculated at the B3LYP/6-311++G(d,p) level of theory. The color gradient on the map spans from −2.3 × 10^−2^ eV (deepest red) to 2.3 × 10^−2^ eV (deepest blue), including regions of varying electrostatic potential.

The molecular electrostatic potential (MEP) has proven to be a valuable tool for investigating the correlation between molecular structure and physicochemical properties [30,31]. The MEP map is a valuable tool for evaluating the reactivity of both inorganic and organic molecules, as well as for identifying potential internal and external molecular interactions. It indicates that areas with the highest positive potential, typically represented in blue, are likely targets for nucleophilic attack. Conversely, areas with the highest negative potential, shown in red, are susceptible to electrophilic attack. When analyzing the MEP map of the two compounds, we observe that the negatively charged regions are largely located over the oxygen atoms of the nitro moiety (Figure 13). These are the sites of strong electrophilic reactivity for the compound. Bromine and chlorine atoms are located in regions characterized by medium electron density, which means that bromine and chlorine are less likely to react either as nucleophiles or electrophiles. On the other hand, the positive regions are mainly surrounding the molecule over the carbons (aromatic CH_3_ for compound **5**) and hydrogen atoms, showing electrophilic reactivity.

### 2.7. Hirshfeld Surface Analyses

The packing of small molecule organic crystals is primarily governed by hydrogen bonding patterns. However, the crystalline structure is the result of multiple forces, necessitating the consideration of all intermolecular interactions. The unique characteristics of crystal packing arise from the molecule’s ability to engage in specific intermolecular interactions. Similarly, the reactivity of compounds is influenced by intermolecular interactions in a reaction medium. Hence, the analysis of intermolecular interactions is crucial. An interesting tool for studying these interactions in the crystalline phase is to investigate Hirshfeld surfaces and 2D fingerprints generated by the CrystalExplorer program [32]. These surfaces were developed to define the molecule’s spatial occupancy within the crystal and facilitate the partitioning of the crystal’s electron density into molecular fragments. Short contacts are visually highlighted in red on Hirshfeld surfaces, whereas blue areas indicate longer distances.

**Figure 14:**
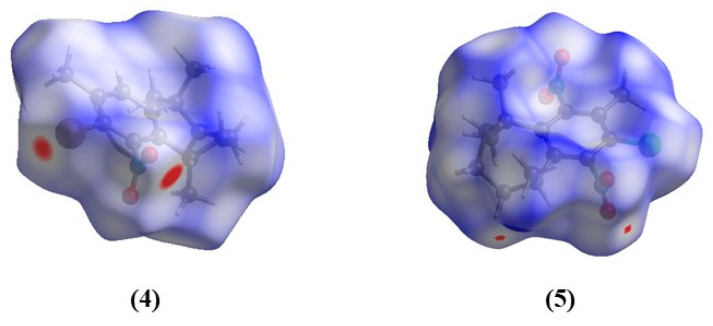
Three-dimensional Hirshfeld surface of compounds **4** and **5** mapped with d_norm_.

**Figure 15:**
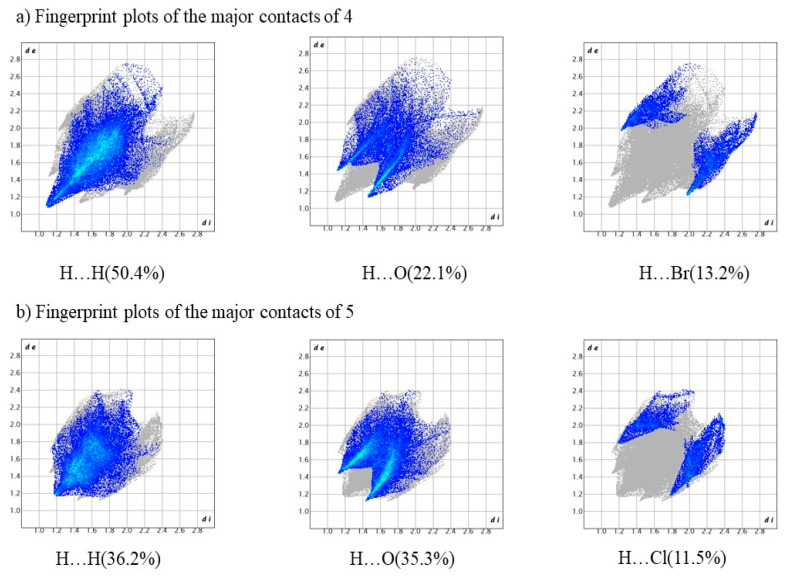
Fingerprint plots of the major contacts of compounds **4** and **5**.

Figure 14 shows the Hirshfeld surfaces plotted over d_norm_ for compounds **4** and **5**, along with the fingerprint plot of their intermolecular contacts shown in Figure 15. The analysis of these surfaces and fingerprint plots reveals significant intermolecular interactions in both crystal packings. In the compound **4** crystal packing, the most prevalent interactions are H…H (50.4%) and H…O (22.1%). Similarly, the compound **5** crystal packing is dominated by H…H interactions at 36.2%, followed by H…O at 35.3% (Figure 15). Additionally, H…Br and H…Cl interactions are present, accounting for 13.2% and 12% of the total Hirshfeld surface, respectively. Importantly, the absence of long sharp spikes indicates the lack of strong hydrogen bonds in the crystal structures of the title compounds (Figure 15).

### 2.8. Molecular Docking Analysis

Molecular docking is an essential method used in computational drug design and screening processes. It enables the evaluation of the interaction between a molecule and a receptor by determining the binding affinity score [33]. In this research, we explored the potential of compounds synthesized from the synthesis of two halogenated nitro derivatives of arylhimachalene: bromo-nitro-arylhimachalene and chloro-dinitro-arylhimachalene, using molecular docking. These compounds exhibited notable efficacy in modulating the activity of assorted isolated smooth muscles and in interactions with diverse neurotransmitters. Docking scores were employed to evaluate the binding affinity strength in ligand–receptor interactions, where a lower (more negative) score signifies a more robust association between the target and ligand (Table 5).

The findings reveal that the bromo-nitro-arylhimachalene and chloro-dinitro-arylhimachalene compounds exhibited significant binding affinities to the active site of protein 7B2W. The estimated binding energies were −6.211 kcal/mol and −6.967 kcal/mol, respectively, which significantly exceeded the binding energy of the positive control, physostigmine, recorded at 3.950 kcal/mol in this research. The consistency between our results and those reported in the literature is notable. In previous studies, himachalone monohydrochloride demonstrated binding energies of −5.798 kcal/mol, trans-himachalol showed −7.137 kcal/mol, and himachalone −7.049 kcal/mol [5]. These findings reinforce the validity of our research outcomes, suggesting that our compounds possess comparable or even superior inhibitory potential. Such consistency across different studies underscores the robustness of our experimental methodology and the reliability of our results.

**Figure 16:**
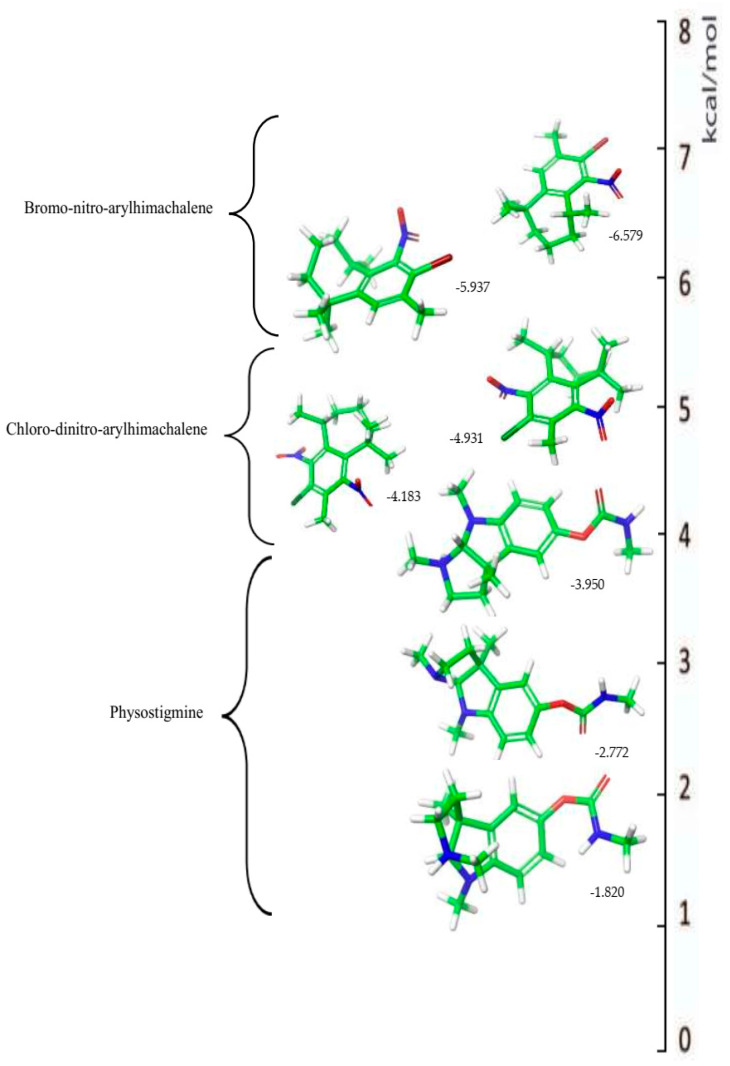
The scale represents the number of conformations obtained for each energy level.

While the molecular docking process commonly faces challenges related to efficient sampling of conformations and orientations, ensuring that ligand conformations maintain low energy levels is occasionally disregarded. However, this aspect is crucial, as high-energy conformations can serve as decoys, potentially masking the presence of more favorable poses [34]. Neglecting electrostatic energies in ligand conformations is one factor that can contribute to such high-energy states. Often, these energies are overlooked due to concerns about their potential overemphasis in calculations employing effectively low dielectric environments [35] (Figure 16).

**Figure 17:**
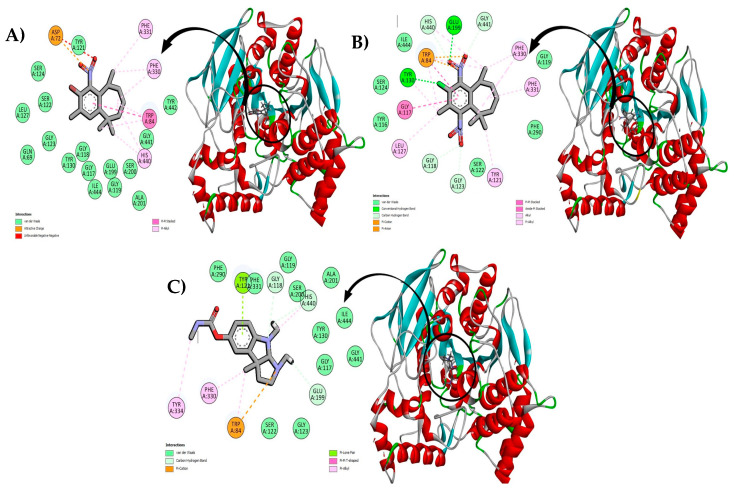
Two-dimensional and three-dimensional representations of intermolecular interactions between (**A**) bromo-nitro-arylhimachalene, (**B**) chloro-dinitro-arylhimachalene, and (**C**) physostigmine (control), with the active site of the protein 7B2W.

Further investigations were conducted to determine the specific interactions of the more stable compounds at the protein’s active site and to clarify the mechanism of inhibition. As depicted in Figure 17, bromo-nitro-arylhimachalene and chloro-dinitro-arylhimachalene, which displayed the lowest binding energy and are considered the most probable active inhibitors compared to the control, interacted with the surrounding residues through various interactions. Experimental validation is essential to assess the therapeutic effects and practical applications of promising compounds. The molecular docking results provide insights, but in vitro and in vivo experiments are necessary for a comprehensive evaluation. These experiments will elucidate interactions with target receptors and enzymes, pharmacokinetics, safety, and efficacy profiles. Such an investigation is crucial for advancing the halogenated nitro derivatives of arylhimachalene as potential drugs for medical treatments [36].

## 3. Materials and Methods

### 3.1. Materials

All chemicals and solvents were purchased from Sigma-Aldrich and were utilized as received, without additional purification. The ^1^H and ^13^C NMR spectra were acquired using a Bruker Avance 300 spectrometer operating at 300 MHz for ^1^H and 75 MHz for ^13^C (University of Colorado Boulder, Boulder, CO, USA), with tetramethylsilane serving as the internal standard. Chemical shifts were reported in δ values (ppm). X-ray diffraction analyses were performed on a Bruker D8 VENTURE Super DUO diffractometer (Bruker, Billerica, MA, USA).

### 3.2. Procedure for the Synthesis of Compound ***2***

In a 100 mL round-bottom flask equipped with a magnetic stirrer, 1 g (4.901 mmol) of arylhimachalene and 1.42 g (17.5 mmol) of HBr were introduced. Then, 0.5 mL of H_2_O_2_ was added dropwise over 1 h. The reaction mixture was stirred for 24 h at 0 °C. At the end of the reaction, monitored by thin-layer and gas chromatography, the mixture was neutralized by a saturated aqueous solution of NaHCO_3_, extracted with ethyl acetate (3 × 20 mL). The organic layers were gathered and dried over anhydrous magnesium sulfate (MgSO_4_) and filtered. The solvent was removed in vacuo. The residue was purified by column chromatography using silica gel with hexane/AcOEt (98:2) as the eluent.

Yield = 87%. Yellow oil. NMR ^1^H (300 MHz, CDCl_3_, δ ppm): 7.23 (1 H, s, ArCH); 7.11 (1 H, s, ArCH); 3.12 (1 H, m, CH-CH_3_); 2.25 (3 H, s, CH_3_-Ar); 1.66 (4 H, m, CH_2_); 1.46 (2 H, m, CH_2_); 1.23 (3 H, s, CH_3_); 1.24 (3 H, s, CH_3_); 1.22 (3 H, s, CH_3_).

NMR ^13^C (75 MHz, CDCl_3_, δ ppm): 147.3; 144.1; 134.6; 129.4; 129.3; 122.3; 40.9; 39.3; 36.3; 34.4; 33.9; 29.5; 24.1; 22.5; 20.9.

### 3.3. Procedure for the Synthesis of Compound ***3***

A mixture of arylhimachalene (1 g, 4.9 mmol) and 2 equivalents of N-chlorosuccinimide (NCS) in acetonitrile (10 mL) was stirred at 80 °C for 24 h. At the end of the reaction, monitored by thin-layer and gas chromatography, the mixture was extracted with dichloromethane (3 × 20 mL). Then, the organic layer was dried over anhydrous magnesium sulfate (MgSO_4_) and filtered. The solvent was removed in vacuo. The residue was purified by column chromatography using silica gel with hexane/AcOEt (98:2) as the eluent.

Yield: 85%, colorless oil, NMR ^1^H (300 MHz, CDCl_3_, δ ppm): 7.09 (1 H, s, Ar-CH); 7.04 (1H, s, Ar-CH); 3.11 (1H, m, CH-CH_3_); 2.21 (3H, s, CH_3_-Ar); 1.64 (4H, m, CH2); 1.41 (2H, m, CH_2_); 1.29 (3H, s, CH_3_); 1.22 (3H, s, CH_3_); 1.20 (3H, s, CH_3_).

NMR ^13^C (75 MHz, CDCl3, δ ppm): 146.4; 143.7; 132.6; 131.7; 129.4; 126.1; 41.1; 39.3; 36.4; 34.4; 34.1; 29.6; 24.1; 20.9; 19.7.

### 3.4. Procedure for the Synthesis of Compounds ***4*** and ***5***

In a 100 mL three-necked round-bottom flask equipped with a magnetic stirrer, a dropping funnel, and a condenser, 1 g of (compound **2**) or (compound **3**) was introduced, followed by 4 mL of H_2_SO_4_. In an Erlenmeyer flask, a mixture of 1.2 mL of HNO_3_ and 1.2 mL of H_2_SO_4_ was prepared and added to the dropping funnel. Then, the acid mixture was added dropwise to the reaction mixture within 1 h 30 min at 0 °C. The solution was then stirred for 12 h at room temperature. After completion of the reaction, 20 mL of a saturated NaHCO_3_ solution was added to the reaction mixture, then extracted with ethyl acetate, washed with water, and dried over MgSO_4_. After the evaporation of the solvent, the crude product was purified by chromatography on silica gel with hexane/ethyl acetate (90:10) as the eluent. The recrystallization of the products was performed by slow evaporation from a hexane solution.

2-bromo-3,5,5,9-tetramethyl-1-nitro-6,7,8,9-tetrahydro-5H-benzo[7]annulene (Compound **4**)

Yield: 83%, colorless crystals, NMR ^1^H (300 MHz, CDCl_3_, δ ppm): 7.19 (1H, s, H-Ar), 3.24 (1H, m, CH-CH_3_), 2.22 (3H, s, Ar-CH_3_), 1.77 (4H, m, CH_2_), 1.44 (2H, m, CH-CH_2_), 1.41 (3H, s, CH_3_), 1.35 (3H, d, J = 7.31, CH_3_), 1.32 (3H, s, CH_3_).

NMR ^13^C (75 MHz, CDCl_3_, δ ppm): 154.08; 142.14; 138.34; 132.03; 130.72; 114.87; 42.79; 41.15; 35.28; 33.57; 29.58; 27.28; 20.60; 20.07; 19.87.

2-chloro-3,5,5,9-tetramethyl-1,4-dinitro-6,7,8,9-tetrahydro-5H-benzo[7]annulene (Compound **5**)

Yield: 79%, colorless crystals, NMR ^1^H (300 Mhz, CDCl_3_, δ ppm): 3.24 (1H, m, CH-CH_3_), 2.24 (3H, s, Ar-CH_3_), 1.75 (4H, m, CH_2_), 1.52 (2H, m, CH-CH_2_), 1.40 (3H, s, CH_3_), 1.34 (3H, d, J = 7.32, CH_3_), 1.31 (3H, s, CH_3_).

NMR ^13^C (75 MHz, CDCl_3_, δ ppm): 154.21, 145.21, 141.78, 138.64, 130.68, 114.89, 42.94, 41.38, 35.29, 33.48, 29.70, 27.39, 20.08, 19.84

### 3.5. Crystal Structure Determination

Single-crystal X-ray diffraction measurements of the two synthesized products were carried out at room temperature using a Bruker D8 VENTURE Super DUO diffractometer [37] equipped with Cu-Kα radiation (λ = 1.5418 Å) at 173 K (Table 6). The structure was solved by direct methods and successive Fourier difference synthesis using SHELXS 2014 [38] and refined by the full-matrix least-squares procedure on F2. The positions of non-hydrogen atoms were refined anistropically by SHELXL-2014 [39]. Hydrogen atoms were placed in chemically acceptable positions. The ORTEP of the compound was generated using the ORTEP3 [40], and the packing diagram was generated using Mercury software [41]. A total of 351 parameters were refined with 5862 unique reflections, which covered the residuals to R1 0.054. Crystallographic data were deposited in the Cambridge Crystallographic Data Centre as supplementary publications with CCDC numbers 2303321 and 2303322. Copies of the data can be obtained free of charge via http://www.ccdc.cam.ac.uk/conts/retrieving.html or from the Cambridge Crystallographic Data Centre, 12 Union Road, Cambridge CB2 1EZ, UK by fax: 044 1223 336 033 or e-mail: deposit@ccdc.cam.ac.uk.

### 3.6. Computational Method

Full geometry optimizations of the arylhimachalene derivative substituted with bromine and chlorine were carried out using the Gaussian09 software package [43], employing the density functional theory (DFT) method. All calculations were performed utilizing the B3LYP hybrid density functional theory with the 6-31++G(d,p) basis set. Vibrational frequency calculations were conducted to verify that the optimized geometries represent local minima on the potential energy surfaces (PESs). The absence of imaginary frequencies confirmed that the stationary points corresponded to minima on the total PESs.

From the DFT calculation and after confirming the optimized structures, electronic properties related to the frontier molecular orbital energies of the highest occupied molecular orbital (HOMO) and the lowest unoccupied molecular orbital (LUMO) were theoretically determined at the same level. Several local reactivity indices, such as chemical potential (μ), chemical hardness (η), electronegativity (χ), and electrophilicity index (ω), were calculated. These descriptors helped to understand the structure of the molecules and their reactivity. To gain further insights into the electrophilic and nucleophilic sites as well as hydrogen-bonding interactions in the synthesized arylhimachalene, the molecular electrostatic potential (MEP) was calculated. Additionally, the intermolecular interaction analysis of the crystal structure was determined through the Hirschfeld surface plots generated by using the Crystal Explorer [32].

### 3.7. In Silico Molecular Docking

#### 3.7.1. Ligand Preparation

Two halogenated nitro derivatives of arylhimachalene, namely bromo nitro-arylhimachalene and chloro-dinitro-arylhimachalene, which have been reported as dual inhibitors, were sketched using Maestro 12.8 (Schrodinger, LLC, New York, NY, USA) [44]. A physostigmine inhibitor was employed as the standard (positive control) in this study. Each structure was assigned an appropriate bond order using the LigPrep package from Schrodinger [45]. The inhibitors were then converted to SDF (Structures Data File) using Maestro and optimized using the optimized potentials for liquid simulations (OPLS 2005) force field with default settings [46].

#### 3.7.2. Protein Structure Preparation

In this investigation, the structure of the Torpedo californica acetylcholinesterase complexed with UO2 (PDBID: 7B2W) was obtained from the RCSB protein data bank (RCSB PDB) [5]. The PDB structure contains several instances of missing information regarding specific connectivity, bond orders, and formal charges. The protein structure was imported from the PDB into Maestro using the protein preparation wizard. Within this wizard, the option to display only polar hydrogen atoms was selected [47]. This choice allows for the representation of solely polar hydrogen atoms in the structure [48].

#### 3.7.3. Grid-Based Ligand Docking with Energetic

Grid-based lLigand dDocking with eEnergetic (GLIDE), a component of the Schrödinger suite, was utilized for docking analysis. It explores favorable interaction regions between ligands and proteins, employing a hierarchical series of filters to locate specific positions of the ligand within the protein’s active site [49]. GLIDE SP (standard precision) mode, designed for high-quality ligand poses, exclusively docks the top-scoring ligands. The docking process involves protein structure preparation, receptor grid generation, ligand structure preparation, and ligand docking. Subsequently, the chosen conformation is depicted in a 2D and 3D diagram, illustrating the ligand’s interactions with active site residues using BIOVIA Discovery Studio 2021 [50].

## 4. Conclusions

We report the synthesis of two halogenated nitro derivatives of arylhimachalene: 2-bromo-3,5,5,9-tetramethyl-1-nitro-6,7,8,9-tetrahydro-5H-benzocycloheptene (bromo-nitro-arylhimachalene) and 2-cloro-3,5,5,9-tetramethyl-1,3-dinitro-6,7,8,9-tetrahydro-5H-benzocycloheptene (chloro-dinitro-arylhimachalene). These compounds were synthesized through the chlorination and bromination of arylhimachalene, followed by the nitration of the halogenated intermediates using straightforward and efficient methods. The compounds were characterized by ^1^H and ^13^C NMR spectrometry and X-ray structural analysis. Density functional theory calculations were conducted, revealing a strong correlation with the experimental data. Furthermore, the frontier molecular orbitals, reactivity parameters, and molecular electrostatic potential were evaluated. Hirshfeld surface and 2D fingerprint analyses showed significant intermolecular interactions, predominantly H–H and H–O contacts. The molecular docking results demonstrate the interaction of the synthesized compounds with receptors, suggesting their potential for therapeutic application optimization.

## Figures and Tables

**Table 1:** Hydrogen bond geometry (Å, °).

D—H···A	D—H	H…A	D···A	D—H···A
C30—H30A···O2^i^	0.98	2.675	3.557 (11)	166
C4—H4B···Cg1^ii^	0.98	2.82	3.67 (14)	144

Symmetry codes: (i) −x + 1 and y + 1/2, −z + 3/2; (ii) −x, y + 3/2, and −z + 3/2.

**Table 2:** Theoretical and experimentally selected structural parameters of compounds **4** and **5**.

	Compound 4		Compound 5	
Bond Length Å	Calculated	Experimental	Calculated	Experimental
C1–C14	1.421	1.432	1.433	1.429
C1–C19	1.410	1.375	1.408	1.375
C18–C19	1.390	1.402	1.389	1.384
C17–C32	1.506	1.572	1.511	1.518
C18–X39	1.904	1.891	1.747	1.729
C19–N36	1.487	1.481	1.489	1.489
C13–C14	1.559	1.527	1.575	1.563
C1–C2	1.537	1.522	1.545	1.542
**Bond angles °**	**Calculated**	**Experimental**	**Calculated**	**Experimental**
C1–C14–C15	117.6	116.5	114.9	114.8
C1–C19–C18	124.9	125.8	125.3	125.8
C1–C14–C13	127.2	126.5	124.7	124.2
C1–C2–C4	113.2	112.1	114.7	115.3
C19–C18–X39	120.7	120.7	120.2	120.2
C1–C19–N36	118.7	120.2	119.7	119.8
C15–C17–C32	121.1	123.3	123.4	123.5
C14–C13–C10	114.8	114.8	113.3	111.7
**Torsion Angles °**	**Calculated**	**Experimental**	**Calculated**	**Experimental**
C1–C19–C18–X39	178.7	177.2	178.8	178.6
N36–C19–C18–X39	−2.5	−4.9	−2.7	−4.7
C19–C1–C2–C4	−139.8	−141.2	−141.4	−147.0
C1–C14–C13–C10	19.0	23.2	22.7	24.0

**Table 3:** Calculated chemical parameters (eV), energy gap (Egap), electronic chemical potential (μ), chemical hardness (η), electronegativity (χ), and electrophilicity index (ω) of compounds **4** and **5** obtained from the DFT/B3LYP method.

Compound	HOMO	LUMO	Egap	μ	η	χ	ω
**4**	−6.878	−2.483	4.396	−4.681	4.396	4.681	2.492
**5**	−7.485	−2.959	4.526	−5.222	4.526	5.222	3.013

**Table 4:** Energy (eV) and composition of some selected molecular orbitals of compounds **4** and **5**.

MO	Compound 4			Compound 5		
	Energy	Center	Composition %	Energy	Center	Composition %
LUMO+1	−1.090	C1	0.663	−2.838	C1	4.926
		C14	26.414		C14	7.112
		C15	16.379		C15	17.423
		C17	1.886		C17	7.528
		C18	28.515		C18	18.456
		C19	17.417		N36	5.528
		N36	0.671		O37	3.459
		O37	0.605		O38	3.620
		O38	0.566		N39	11.464
		Br39	2.274		O40	6.397
					O41	6.719
LUMO	−2.483	C1	1.517	−2.959	C14	6.848
		C18	1.333		C15	7.539
		C19	0.927		C18	7.677
		N36	43.969		N39	30.608
		O37	25.017		O40	16.589
		O38	24.730		O41	18.698
HOMO	−6.878	C1	18.491	−7.485	C1	21.019
		C14	22.213		C14	21.773
		C17	15.137		C16	17.859
		C18	19.209		C17	17.979
		Br39	15.753		Cl35	9.525
HOMO-1	−7.197	C1	8.902	−7.644	C1	6.988
		C14	4.001		C14	4.226
		C15	30.371		C15	28.280
		C17	12.600		C16	7.567
		C18	2.057		C17	4.590
		C19	28.718		C18	20.565
		O37	1.807		Cl35	4.359
		O38	1.946		O37	2.560
		Br39	3.655		O38	2.375
					O10	2.421
					O41	2.015

**Table 5:** The docking scores (kcal/mol) and detailed interaction studies of the selected compounds against the protein 7B2W.

Compound	Docking Score	Interacting Amino Acid	Interaction Type
Bromo-nitro-arylhimachalene	−6.579	ASP72	Attractive charge
		TRP84	Pi–Pi Stacked
		PHE330, PHE331, HIS440, TRP84	Pi–Alkyl
		TYR121, SER124, SER122, LEU127, GLN69, GLY123, TYR130, GLY118, GLY117, ILE444, GLU199, GLY119, SER200, ALA201, GLY441, TYR442	Van der Waals
Chloro-dinitro-arylhimachalene	−4.931	GLU199, TYR130	Conventional hydrogen bond
		HIS440, GLY441, GLY118, GLY123	Carbon–hydrogen bond
		GLY117	Amide–Pi Stacked
		TRP84	Pi–cation
		TRP84	Pi–anion
		TRP84	Pi–Pi Stacked
		LEU127	Alkyl
		TYR121, PHE331, PHE330, HIS440, TRP84, TYR130	Pi–Alkyl
		ILE444, SER124, TYR116, PHE290, SER122, GLY119	Van der Waals
Physostigmine	−3.950	TRP84	Pi–cation
		TYR121	Pi–lone pair
		HIS440	Pi–Pi T-shaped
		GLU199, GLY118, HIS440	Carbon–hydrogen bond
		TYR334, PHE330, HIS440	Pi–Alkyl
		PHE290, PHE331, GLY119, SER200, ALAL201, TYR130, ILE444, GLY117, GLY441, GLY123, SER122	Van der Waals

**Table 6:** Experimental X-ray data collection from the synthesized products.

	C_15_H_20_BrNO_2_ (Compound 4)	C_15_H_19_ClN_2_O_4_ (Compound 5)
M_r_	326.23	326.77
Crystal system, space group	Orthorhombic, P212121	Orthorhombic, P212121
Temperature (K)	296	173
a, b, c (Å)	10.2548 (5), 15.6927 (8), 19.1418 (9)	9.4448 (3), 9.5590 (3), 16.6190 (6)
V (Å^3^)	3080.4 (3)	1500.41 (9)
Z	8	4
Radiation type	Cu Kα (λ = 1.54178 Å)	Cu Kα (λ = 1.54178 Å)
m (mm^−1^)	3.62	2.44
Crystal size (mm)	0.32 × 0.24 × 0.19	0.31 × 0.26 × 0.21
Data collection		
Diffractometer	Bruker D8 VENTURE Super DUO	Bruker D8 VENTURE Super DUO
Absorption correction	Multi-scan	Multi-scan
	SADABS (Krause et al., 2015 [42])	SADABS (Krause et al., 2015 [42])
T_min_, T_max_	0.616, 0.747	0.539, 0.753
No. of measured, independent and	46,768, 5879, 4972	31,539, 2851, 2728
observed [I > 2s(I)] reflections		
Rint	0.054	0.049
(sin q/l) max (Å^−1^)	0.611	0.606
Refinement		
R[F^2^ > 2s(F^2^)], wR(F^2^), S	0.054, 0.167, 1.03	0.028, 0.074, 1.06
No. of reflections	5862	2851
No. of parameters	351	205
No. of restraints	102	0
H-atom treatment	H-atom parameters constrained	H-atom parameters constrained
Dρmax, Dρmin (e Å^−3^)	0.64, −0.52	0.21, −0.21
Absolute structure	Flack x determined using 1918 quotients [(I+) − (I−)]/[(I+) + (I−)] (Parsons, Flack and Wagner, ActaCryst. B69 (2013) 249–259)	Refined as an inversion twin
Absolute structure parameter	0.061 (13)	0.065 (17)

## Data Availability

The data presented in this study are available upon request from the corresponding author.

## References

[1] Aberchane M., Fechtal M., Chaouch A. (2004). Analysis of Moroccan Atlas Cedarwood Oil (*Cedrus atlantica* Manetti). J. Essent. Oil Res..

[2] Abouhamza B., Allaoud S., Karim A. (2001). Ar-Himachalene. Molecules.

[3] Geha R.S., Meltzer E.O. (2001). Desloratadine: A New, Nonsedating, Oral Antihistamine. J. Allergy Clin. Immunol..

[4] Bryson H.M., Wilde M.I. (1996). Amitriptyline. A Review of Its Pharmacological Properties and Therapeutic Use in Chronic Pain States. Drugs Aging.

[5] Faris A., Edder Y., Louchachha I., Lahcen I.A., Azzaoui K., Hammouti B., Merzouki M., Challioui A., Boualy B., Karim A. (2023). From Himachalenes to Trans-Himachalol: Unveiling Bioactivity through Hemisynthesis and Molecular Docking Analysis. Sci. Rep..

[6] Chaudhary A., Kumar N., Kumar R., Salar R.K. (2019). Antimicrobial Activity of Zinc Oxide Nanoparticles Synthesized from Aloe Vera Peel Extract. SN Appl. Sci..

[7] Chaudhary A., Das P., Mishra A., Kaur P., Singh B., Goel R.K. (2012). Naturally Occurring Himachalenes to Benzocycloheptene Amino Vinyl Bromide Derivatives: As Antidepressant Molecules. Mol. Divers..

[8] Yamini Y., Anand P., Bhardwaj V.K., Kumar A., Purohit R., Das P., Padwad Y. (2023). Novel Pyrrolone-Fused Benzosuberene MK2 Inhibitors: Synthesis, Pharmacophore Modelling, Molecular Docking, and Anti-Cancer Efficacy Evaluation in HNSCC Cells. J. Biomol. Struct. Dyn..

[9] Zollinger H. (1989). Color Chemistry: Synthesis, Properties and Applications of Organic Dyes and Pigments. Leonardo.

[10] Paden N.E., Smith E.E., Maul J.D., Kendall R.J. (2011). Effects of Chronic 2,4,6,-Trinitrotoluene, 2,4-Dinitrotoluene, and 2,6-Dinitrotoluene Exposure on Developing Bullfrog (*Rana catesbeiana*) Tadpoles. Ecotoxicol. Environ. Saf..

[11] Becker F.F., Banik B.K. (1998). Polycyclic Aromatic Compounds as Anticancer Agents: Synthesis and Biological Evaluation of Some Chrysene Derivatives. Bioorg. Med. Chem. Lett..

[12] Sana S., Tasneem, Ali M.M., Rajanna K.C., Saiprakash P.K. (2009). Efficient and Facile Method for the Nitration of Aromatic Compounds by Nitric Acid in Micellar Media. Synth. Commun..

[13] Bak R.R., Smallridge A.J. (2001). A Fast and Mild Method for the Nitration of Aromatic Rings. Tetrahedron. Lett..

[14] Cheng G., Duan X., Qi X., Lu C. (2008). Nitration of Aromatic Compounds with NO_2_/Air Catalyzed by Sulfonic Acid-Functionalized Ionic Liquids. Catal. Commun..

[15] Zoubir M., Belghiti M.E., Idrissi M.E., Zeroual A. (2022). Theoretical Investigation of the Mechanism and Regioselectivity of the 3-Isopropyl-1,6-Dimethyl-Naphthalene and Ar-Himachalene in Nitration Reaction: A MEDT Study. Theor. Chem. Acc..

[16] Buu-Hoï N.P. (1954). Quelques Nouvelles Halogénations Effectuées Avec Les N-bromo- et N-chlorosuccinimides. Recl. Des Trav. Chim. Des Pays.-Bas..

[17] Tanemura K., Suzuki T., Nishida Y., Satsumabayashi K., Horaguchi T. (2003). Halogenation of Aromatic Compounds by N-Chloro-, N-Bromo-, and N-Iodosuccinimide. Chem. Lett..

[18] Salama T.A., Novák Z. (2011). N-Halosuccinimide/SiCl4 as General, Mild and Efficient Systems for the α-Monohalogenation of Carbonyl Compounds and for Benzylic Halogenation. Tetrahedron. Lett..

[19] Gu L., Lu T., Zhang M., Tou L., Zhang Y. (2013). Efficient Oxidative Chlorination of Aromatics on Saturated Sodium Chloride Solution. Adv. Synth. Catal..

[20] Barhate N.B., Gajare A.S., Wakharkar R.D., Bedekar A.V. (1998). Simple and Efficient Chlorination and Bromination of Aromatic Compounds with Aqueous TBHP (or H_2_O_2_) and a Hydrohalic Acid. Tetrahedron. Lett..

[21] Khazaei A., Zolfigol M.A., Kolvari E., Koukabi N., Soltani H., Bayani L.S. (2010). Electrophilic Bromination of Alkenes, Alkynes, and Aromatic Amines with Iodic Acid/Potassium Bromide under Mild Conditions. Synth. Commun..

[22] Krishna Mohan K.V.V., Narender N., Kulkarni S.J. (2004). Simple and Regioselective Oxyiodination of Aromatic Compounds with Ammonium Iodide and Oxone^®^. Tetrahedron. Lett..

[23] Gruter G.J.M., Akkerman O.S., Bickelhaupt F. (1994). Nuclear versus Side-Chain Bromination of Methyl-Substituted Anisoles by N-Bromosuccinimide. J. Org. Chem..

[24] Edder Y., Lahcen I.A., Brahim H., Boualy B., Karim A., Pérez-Redondo A. (2019). Synthesis, Crystal Structure and Spectral Characterization of (Z)-2,8-Dibromo-9-(Bromomethylene)-3,5,5-Trimethyl-6,7,8,9-Tetrahydro-5H-Benzo[7]Annulene. J. Mol. Struct..

[25] Spek A.L. (1990). PLATON, an Integrated Tool for the Analysis of the Results of a Single Crystal Structure Determination. PLATON Integr. Tool Anal. Results A Single Cryst. Struct. Determ..

[26] Cremer D., Pople J.A. (1975). A General Definition of Ring Puckering Coordinates. J. Am. Chem. Soc..

[27] Flack H.D., Bernardinelli G. (2000). Reporting and Evaluating Absolute-Structure and Absolute-Configuration Determinations. J. Appl. Crystallogr..

[28] Bakhouch M., El Yazidi M., Al Houari G., Saadi M., El Ammari L. (2017). 3′-(4-Chlorophenyl)-4′-Phenyl-3 *H*,4′ *H*-Spiro[Benzo[*b*]Thiophene-2,5′-Isoxazol]-3-One. IUCrdata.

[29] Koopmans T. (1934). Über Die Zuordnung von Wellenfunktionen Und Eigenwerten Zu Den Einzelnen Elektronen Eines Atoms. Physica.

[30] Luque F.J., López J.M., Orozco M. (2000). Perspective on “Electrostatic Interactions of a Solute with a Continuum. A Direct Utilization of Ab Initio Molecular Potentials for the Prevision of Solvent Effects”. Theor. Chem. Acc..

[31] Chidangil S., Shukla M.K., Mishra P.C. (1998). A Molecular Electrostatic Potential Mapping Study of Some Fluoroquinolone Anti-Bacterial Agents. J. Mol. Model..

[32] Turner J., McKinnon J.J., Wolff S.K., Grimwood D.J., Spackman P.R., Jayatilaka D., Spackman M.A. (2017). CrystalExplorer17.

[33] Wihadi M.N.K., Merzouki M., Ma’arif A.S., Grasianto G., Santosa S.J. (2024). Insights of Auric Ion Adsorption in the Presence of Ferric and Hexavalent Chromium Species on Mg/Al Layered Double Hydroxides. Moroc. J. Chem..

[34] Merzouki M., Bourassi L., Abidi R., Bouammali B., El Farh L., Sabbahi R., Challioui A. (2024). Deciphering the SARS-CoV-2 Delta Variant: Antiviral Compound Efficacy by Molecular Docking, ADMET, and Dynamics Studies. Moroc. J. Chem..

[35] Coleman R.G., Carchia M., Sterling T., Irwin J.J., Shoichet B.K. (2013). Ligand Pose and Orientational Sampling in Molecular Docking. PLoS ONE.

[36] Bekkouch A., Merzouki M., El Mostafi H., Elhessni A., Challioui A., Mesfioui A., Touzani R. (2024). Potential Inhibition of ALDH by Argan Oil Compounds, Computational Approach by Docking, ADMET and Molecular Dynamics. Moroc. J. Chem..

[37] Bruker (2016). APEX2 (Version 5.054), SAINT (Version 6.36A), SADABS.

[38] Sheldrick G.M. (2008). A Short History of SHELX. Acta Crystallogr. A.

[39] Sheldrick G.M. (2015). Crystal Structure Refinement with SHELXL. Acta Crystallogr. C Struct. Chem..

[40] Farrugia L.J. (2012). WinGX and ORTEP for Windows: An Update. J. Appl. Crystallogr..

[41] Macrae C.F., Bruno I.J., Chisholm J.A., Edgington P.R., McCabe P., Pidcock E., Rodriguez-Monge L., Taylor R., Van De Streek J., Wood P.A. (2008). Mercury CSD 2.0—New Features for the Visualization and Investigation of Crystal Structures. J. Appl. Crystallogr..

[42] Krause L., Herbst-Irmer R., Stalke D. (2015). An empirical correction for the influence of low-energy contamination. J. Appl. Crystallogr..

[43] Frisch M.J., Trucks G.W., Schlegel H.B., Scuseria G.E., Robb M.A., Cheeseman J.R., Scalmani G., Barone V., Petersson G.A., Nakatsuji H. (2016). Gaussian 09, Revision A.02. https://gaussian.com/g09citation/.

[44] Ezekiel O.A., Oluwaotbiloba A.A., Samuel B.A., Oluwatosin A.G., Mary M.N., Rasheedat F.T., Yetunde K.O., Victory O.U., Damilola B.S., Ewele O.G. (2023). Application of In-Silico Methodologies in Exploring the Antagonistic Potential of Trigonella Frenum-Graecum on Cyclooxygenase-2 (Cox-2) in Cancer Treatment. IPS J. Mol. Docking Simul..

[45] Adekiya T.A., Aruleba R.T., Klein A., Fadaka A.O. (2022). In Silico Inhibition of SGTP4 as a Therapeutic Target for the Treatment of Schistosomiasis. J. Biomol. Struct. Dyn..

[46] Doherty B., Zhong X., Gathiaka S., Li B., Acevedo O. (2017). Revisiting OPLS Force Field Parameters for Ionic Liquid Simulations. J. Chem. Theory Comput..

[47] Fajriyah N.N., Mugiyanto E., Rahmasari K.S., Nur A.V., Najihah V.H., Wihadi M.N.K., Merzouki M., Challioui A., Vo T.H. (2023). Indonesia Herbal Medicine and Its Active Compounds for Antidiabetic Treatment: A Systematic Mini Review. Moroc. J. Chem..

[48] Bouakline H., Bouknana S., Merzouki M., Ziani I., Challioui A., Bnouham M., Tahani A., El Bachiri A. (2024). The Phenolic Content of Pistacia Lentiscus Leaf Extract and Its Antioxidant and Antidiabetic Properties. Sci. World J..

[49] Powers R., Copeland J.C., Germer K., Mercier K.A., Ramanatlian V., Revesz P. (2006). Comparison of Protein Active Site Structures for Functional Annotation of Proteins and Drug Design. Proteins Struct. Funct. Genet..

[50] Saeed M., Shoaib A., Tasleem M., Alabdallah N.M., Alam M.J., El Asmar Z., Jamal Q.M.S., Bardakci F., Alqahtani S.S., Ansari I.A. (2021). Assessment of Antidiabetic Activity of the Shikonin by Allosteric Inhibition of Protein-Tyrosine Phosphatase 1b (Ptp1b) Using State of Art: An in Silico and in Vitro Tactics. Molecules.

